# Demographic Analysis of Sixteen Iberian Sheep Breeds Based on Pedigree Data

**DOI:** 10.3390/biology15151233

**Published:** 2026-07-24

**Authors:** Laura Marín Marín, Nuno Carolino, María Esperanza Camacho Vallejo, José Ignacio Salgado Pardo, Juan Vicente Delgado Bermejo, José Manuel León Jurado, Esther Díaz Ruiz, Eugenio Espinosa de los Monteros Jaúdenes, José Antonio Puntas Tejero, Manuel Aguilera Ropero, Águeda Laura Pons Barro, José Manuel Alanzor Puente, Sergio Nogales Baena, Claudino Matos, José Rodrigues, Tiago Perloiro, Sara Roque, Maria Inês Carolino, Andreia Vitorino, Antonio González Ariza

**Affiliations:** 1 Department of Genetics, Faculty of Veterinary Sciences, University of Córdoba, 14071 Cordoba, Spain; marinlaura30@gmail.com (L.M.M.); juanviagr218@gmail.com (J.V.D.B.); estherddrr@gmail.com (E.D.R.); 2 Andalusian Institute of Agricultural and Fisheries Research and Training (IFAPA), Alameda del Obispo, 14004 Cordoba, Spain; mariae.camacho@juntadeandalucia.es; 3 Faculdade de Medicina Veterinária, Universidade de Lisboa, Av. Universidade Técnica, 1300-477 Lisbon, Portugal; nuno.carolino@iniav.pt; 4 Instituto Nacional de Investigação Agrária e Veterinária, Polo de Inovação da Fonte Boa—Estação Zootécnica Nacional, 2005-424 Santarem, Portugal; ines.carolino@iniav.pt (M.I.C.); andreia.vitorino@iniav.pt (A.V.); 5 Centro de Investigação Interdisciplinar em Sanidade Animal, Faculdade de Medicina Veterinária, Universidade de Lisboa, 1300-477 Lisbon, Portugal; 6 Laboratório Associado para a Ciência Animal e Veterinária, Faculdade de Medicina Veterinária, Universidade de Lisboa, 1300-477 Lisbon, Portugal; 7 Agropecuary Provincial Centre, Diputación de Córdoba, 14071 Córdoba, Spain; jomalejur@yahoo.es; 8 Asociación Española de Criadores de Ovinos Precoces, 06420 Castuera, Spain; eugenioem@aecop.es; 9 Asociación Nacional de Criadores de Ovino Segureño, 18830 Huescar, Spain; pepepuntas@ancos.org; 10 Asociación de Ganaderos Criadores de la Raza Ovina Lojeña del Poniente Granadino, 18300 Loja, Spain; manoloaguileraropero@gmail.com; 11 Institut de Reserca i Formaciò Agroalimentaria de les Illes Balears IRFAP, Conselleria d’Agricultura, Pesca i Alimentació, Govern Illes Balears, 07009 Palma, Spain; apons@irfap.es (Á.L.P.B.); jalanzor@irfap.es (J.M.A.P.); 12 Asociación Andaluza de Criadores de la Raza Ovina Churra Lebrijana, 41720 Los Palacios y Villafranca, Spain; sergionogalesbaena@gmail.com; 13 Associação de Agricultores do Sul, Rua Cidade de São Paulo, Apartado 296, 7801-904 Beja, Portugal; cmatos@acos.pt; 14 Associação Nacional de Criadores de Ovinos da Raça Churra Galega Bragançana, Edifício Casa do Lavrador, Rua Cláudio Mesquita Rosa, s/n, 5300-653 Braganza, Portugal; joserodrigues@acob.org.pt; 15 Associação Nacional de Criadores de Ovinos da Raça Merinos, Tv. João Rosa 1a, 7005-665 Evora, Portugal; tperloiro@ancorme.com; 16 Agrupamento de Produtores da Raça Ovina Mondegueira, 6430-148 Meda, Portugal; secretariatecnicaapromeda@gmail.com

**Keywords:** Iberian sheep breeds, demographic analysis, pedigree completeness, population fragmentation, genetic conservation, generation interval, reproductive patterns, ovine biodiversity

## Abstract

The Iberian Peninsula hosts a rich diversity of sheep genetic resources shaped by contrasting environments, historical management practices, and varying levels of endangerment. This study provides a comprehensive demographic characterisation of 16 Iberian sheep breeds (12 autochthonous and 4 integrated) using complete pedigree records supplied by breeders’ associations. Population structure was evaluated through census composition, population fragmentation, trends in sire and dam registrations, reproductive patterns, gender ratios, pedigree completeness, and generation intervals. Marked differences were observed among breeds. Integrated breeds consistently exhibited the highest pedigree completeness and a more intensive reproductive structure, characterised by fewer breeding animals but a higher number of offspring per parent. In contrast, many endangered Spanish breeds showed incomplete pedigree records, fewer registered sires and dams, unbalanced sex ratios and prolonged use of older breeding animals, indicating greater demographic vulnerability. Portuguese local breeds displayed more complete pedigree records, together with relatively high numbers of offspring per parent and/or extended reproductive use of breeding animals. Most breeds showed generation intervals close to four years, although longer intervals were observed in the most endangered populations. These findings highlight substantial differences in demographic structure and pedigree quality among Iberian sheep breeds, providing a demographic baseline to improve pedigree recording and support future conservation and breeding strategies, while reinforcing the role of breeders’ associations in preserving the genetic diversity.

## Simple Summary

Iberian sheep breeds are an important part of the agricultural and cultural heritage of Spain and Portugal. Many of these breeds are well adapted to local environments, but some have small populations and may be at risk of decline. In this study, we examined the demographic characteristics of 16 Iberian sheep breeds using information recorded by breeders’ associations. We compared population size, breeding practices, age of breeding animals, and the quality of genealogical records. Several endangered populations showed signs of demographic vulnerability, including small numbers of breeding animals, unbalanced use of males and females, and less complete pedigree records. Integrated breeds generally showed highly complete pedigree records and intensive breeding management, whereas many endangered Spanish breeds exhibited incomplete genealogies, fewer breeding animals, unbalanced use of males and females, and extended reproductive lifespans. Portuguese local breeds generally showed more complete pedigree records and demographic patterns indicative of more stable population management. These findings provide valuable information for breeders, conservation organizations, and policymakers. Improving pedigree recording and monitoring breeding populations will help maintain genetic diversity, support sustainable livestock production, and ensure the long-term preservation of Iberian sheep breeds for future generations.

## 1. Introduction

The Iberian Peninsula hosts four well-defined ovine trunks: churro, merino, Iberian, and entrefino [1]. The spatial distribution of these breeds reflects their adaptative preferences, as the remarkable climatic variability of the peninsula has historically required such genetic diversity for efficient sheep production [2]. Understanding how these breeds are distributed according to their adaptive traits naturally leads to the study of their genetic diversity and population structure. The assessment of a breed’s population structure, along with evaluation of reproductivity, genetic diversity and gene flow derived from genealogical data, represents a cornerstone for the effective design and implementation of conservation programs [3].

In the second half of the 20th century, the livestock sector opted to develop animal lines and genotypes selected for their high productive output and efficient feed conversion [4]. However, the intensified use of these highly productive lines, among other factors, has reduced the breeds’ census and genetic diversity (particularly in disadvantaged regions) and adversely affected the development of sustainable production systems based on local breeds, giving rise to problems such as rural depopulation, landscape degradation, and consequent ecological imbalance and biodiversity loss [5,6].

In response, Spain and Portugal submitted their national plans for animal genetic resources in 2009 and 2013, respectively, in which both included specific strategies for characterization, conservation and genetic improvement.

Several initiatives have been implemented to promote the quality and distinctiveness of products derived from these breeds: the Protected Geographical Indication and Protected Designation of Origin certifications and the eco-label [7], which boost production by native breeds.

Increasing consumer demand for high-quality, authentic products produced under sustainable practices that respect animal welfare and the environment continues to drive the growth potential of this market [8].

Not only have the autochthonous breeds evolved, but the integrated breeds have also adapted to the socio-economic needs of society. The integrated breeds considered in this study originate from the Spanish Merino breed and have adapted to the environment of the Iberian Peninsula since their introduction in the 1960s and 1970s, becoming specialised in meat production [9].

As for the production systems in which these breeds are typically reared, these have traditionally been extensive and transhumant [10]. Nevertheless, social, cultural, and economic changes in recent decades have led to significant transformations in production systems, with notable ecological impacts. Extensive systems remain predominant in several autochthonous breeds, including OCL, OLO, OMQ, OMN and ORM, whereas semi-extensive management is more common in breeds such as OIB. Other breeds, including OBE, OFL, OIF and OMP, are generally managed under extensive conditions for breeding animals, while lambs are finished under semi-intensive systems. OSE is reared under both extensive and semi-extensive production systems [11]. The fact that animals contribute to land management while grazing, cleaning it, and facilitating agricultural work, is one of the main advantages of extensive systems [2].

In this context, the role of breeders’ associations in conservation and genetic improvement is pivotal, as they implement breeding programs. These programs are recognized tools that describe the actions and agents involved in the conservation and genetic improvement of a livestock breed, requiring professional specialization from the associations’ technicians as well as from geneticists and animal reproduction specialists. Endangered breeds must have a conservation program to try to safeguard the genetic diversity found within the population [12]. Alternatively, the non-endangered status breeds focus their programs on selection schemes [13]. Finally, the situation can be even more dire in animal populations without breeding programmes in place. This situation puts genotypes at serious risk, as there are no management measures being implemented under professional supervision, thereby jeopardizing a breed’s genetic diversity and, consequently, its viability [14].

These associations also serve as an important link between farms, providing coordination and services, and contributing to the digitalization and modernization of the sector [15].

The first step in any biodiversity preservation program or genetic improvement strategy is to understand the demographic and genealogical structure [16]. Population and genetic variability indicators can be extracted from the breeds’ genealogical herdbooks using pedigree analysis. Genetic characterization by pedigree analysis makes it possible to define the structure and dynamics of a population over time, considering it as a group of individuals in permanent renewal and considering its gene pool [14].

Similar studies on genetic diversity across multiple autochthonous breeds have been done in Spain on local bovine populations [17,18] and in France on other ovine breeds [19]. In addition, specific analyses have been performed on some of the breeds included in our dataset [20,21,22]. However, to the best of our knowledge, no comprehensive comparative demographic analysis has yet been conducted across such a broad set of sheep breeds from both Spain and Portugal using a common pedigree-based approach. Therefore, this study aimed to characterise the population dynamics and demographic structure of sixteen Iberian sheep breeds. By applying a common analytical framework, this study enables direct comparisons among breeds differing in origin, conservation status, production system and breeding programme implementation, providing a demographic baseline for future genetic diversity analyses and conservation strategies.

## 2. Materials and Methods

Through the present study, an observational demographic analysis was conducted using pedigree records from sixteen Iberian sheep breeds in the Iberian Peninsula. The objective of this work was to characterise population structure, reproductive patterns and pedigree quality using genealogical information maintained in the official genealogical herdbooks, a fundamental source of information for genetic diversity assessments. Twelve populations used in the present study are autochthonous breeds: Segureña (OSE; Spain), Lojeña (OLO; Spain), Ibicenca (OIB; Spain), Mallorquina (OMQ; Spain), Roja Mallorquina (ORM; Spain), Menorquina (OMN; Spain), Churra Lebrijana (OCL; Spain), Campaniça (OCA; Portugal), Churra Galega Bragançana Branca (CGBb; Portugal), Churra Galega Bragançana Preta (CGBp; Portugal), Merino Branco (OMB; Portugal) and Churra Mondegueira (OCM; Portugal). On the other hand, four of the breeds studied in this research are catalogued as integrated in Spain: Berrichon du Cher (OBE), Île de France (OIF), Fleischschaf (OFL) and Merino Precoz (OMP). All these breeds are included in the Official Catalogue of Livestock Breeds of Spain [23] and Portugal [8], with several classified as endangered (OLO, OIB, OMQ, ORM, OMN, OCL, as well as all the Portuguese ones) according to current regulations. Breed codes follow the national regulations established in Spain [24], and the same coding system is used for breeds in Portugal, as no official nomenclature is specified in their national regulations [25].

### 2.1. Data Sources and Pedigree Information

Pedigree data were obtained from the genealogical herdbooks maintained by each of the aforementioned breeders’ associations, covering all available records from the beginning of registration through 2023. Records used for this study included the animal’s identification code, sire and dam codes, sex, year of birth and life status (alive or dead).

The complete genealogical dataset was considered the Total Population (TP), encompassing all founders, ancestors and their descendants, while the Reference Population (RP) comprised only living animals and the Base Population (BP) referred to individuals with one or more unknown parents.

### 2.2. Data Quality Control

Before the demographic analyses, pedigree records were subjected to a quality control procedure to identify and correct inconsistencies in the genealogical information. Duplicate records and pedigree inconsistencies (e.g., self-parentage, circular genealogies, incorrect parental sex assignment and parents younger than their offspring) were identified using custom scripts developed in R (v.4.5.1). All identified issues were reviewed and corrected whenever possible. Records with unresolved inconsistencies or insufficient information were excluded from the final dataset before pedigree analyses.

### 2.3. Demographic Parameters

Descriptive analyses of population structure were carried out using Microsoft Excel 365 to generate tables and figures, following the methodology of González-Cano et al. [18], based on parameters such as the number of registered sires and dams per year, parental age at offspring birth and average offspring per parent. Likewise, the study included the sex ratio of breeding animals and the foundation year of each breeders’ association in relation to the population census (whole, reference, and base populations).

The number of active breeders in 2023, along with the RP, was also considered to assess the degree of farm fragmentation for each breed. The number of active breeders in 2023 was obtained from government sources [21] or, in the case of Portuguese breeds, official reports developed by Carolino et al. [26,27,28,29]. For OMB, the most recent report available dates from 2020; consequently, the number of active breeders recorded in 2024 in the SPREGA database (https://www.sprega.com.pt; accessed on 29 June 2026) was used instead [30].

Using this information, population fragmentation for each breed was quantified as the ratio between the number of animals in the RP and the number of active breeders, corresponding to the number of farms. Thus, the average farm size was computed.

a. Registered sires and dams

The number of registered sires and dams was used to illustrate population trends by sex. For consistency, data from 1990 onwards were considered, although some genealogical books include records dating back to the 1970s for integrated breeds. Limiting the dataset to this period enabled comparisons among breeds within a common timeframe.

b. Parental age at reproduction

The age of sires and dams at the birth or registration of their offspring was analysed, as this parameter is directly related to generation intervals.

c. Reproductive performance

The average number of offspring per sire and per dam was used as an indicator of reproductive performance.

d. Sex ratio

The sex ratio of breeding animals was calculated annually to determine the number of dams per sire (females:males). This parameter was used as an indicator of sire breeding efficiency within each population.

### 2.4. Pedigree Analysis

Generation interval (L) and pedigree completeness (PC) were estimated for both the total population (TP) and the reference population (RP) using scripts in R (v.4.5.1) and the ENDOG software v.4.8 [31], respectively.

a. Generation Interval (L)
  L was computed through the four paths of selection (father–son, father–daughter, mother–son, and mother–daughter) following the procedure described by Lacy [32]. The average L for each population was defined as the mean of the four pathways.
  L can be computed using ENDOG software; however, overflow errors occurred during the analysis of this parameter for the RP of OIB, ORM, and OCM. Hence, the calculations were performed using R scripts following the approach described by Pierini et al. [33].
b. Pedigree completeness (PC)
  The pedigree completeness (PC) is defined as the proportion of known ancestors in each ascending generation [34]. For each breed, ENDOG computes:
  1. The number of fully traced generations, defined as the number of generations back to the furthest ancestral generation where every single theoretical ancestor is known. A “fully traced” generation requires all 2 g ancestors in that specific generation to be identified (where g is the generation number).
  2. The maximum number of generations traced, defined as the number of generations separating the individual from its furthest ancestor.

The equivalent complete generations, computed as the sum over all known ancestors of the terms, being this the sum of (1/2)*n*, where *n* is the number of generations separating the individual from each known ancestor, for each animal in the pedigree data [31,35].

## 3. Results

### 3.1. Distribution of the Breeds

**Figure 1:**
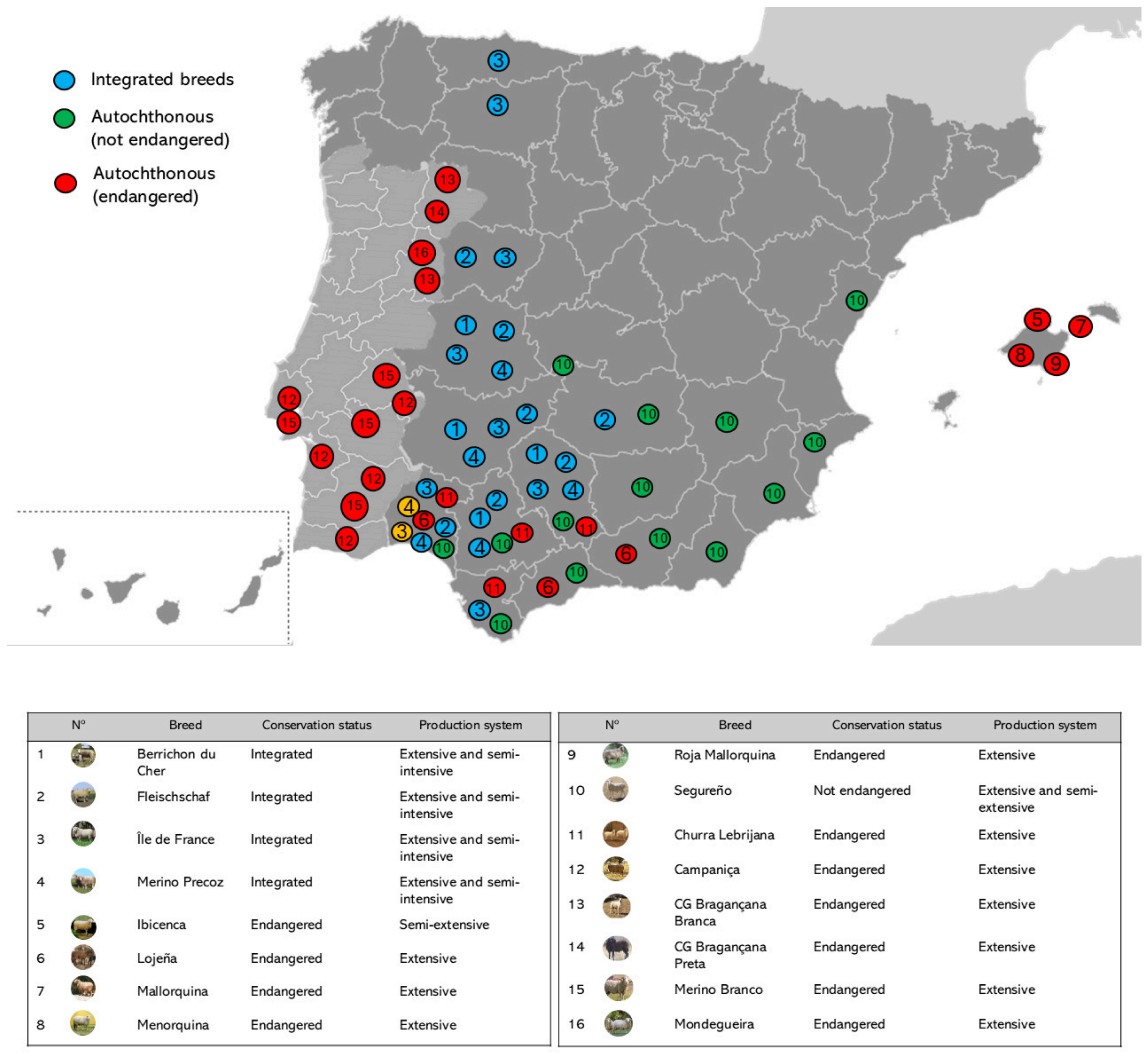
Geographical distribution of Berrichon du Cher (1), Fleischschaf (2), Île de France (3), Merino Precoz (4), Ibicenca (5), Lojeña (6), Mallorquina (7), Menorquina (8), Roja Mallorquina (9), Segureña (10), Churra Lebrijana (11), Campaniça (12), Churra Galega Bragançana Branca (13), Churra Galega Bragançana Preta (14), Merina Branca (15) and Churra Mondegueira (16) sheep breeds in Spain.

As shown in Figure 1, local breeds are predominantly concentrated in specific regions of the Iberian Peninsula. OIB, OMQ, ORM, and OMN are restricted to the Balearic Islands, and OLO and OCL are only found in the mountainous area of Loja and in the marismas (wetlands) of Huelva and Seville, in southern Spain, respectively. CGBb and CGBp share territories in northern Portugal, in Bragança, whereas OCM, OMB, and OCA are arranged along a north–south gradient across Portugal, with OCM in Terra Fria Beira, OMB from the Central to the Algarve area, and OCA in the Alentejo and Costa Vicentina area (southern Portugal). These breeds occupy very limited geographic areas compared with the broader distribution of integrated breeds across the Peninsula, except for OSE, which extends over a large part of the territory.

### 3.2. Breed Census and Foundation Year of Breeders’ Associations

**Figure 2:**
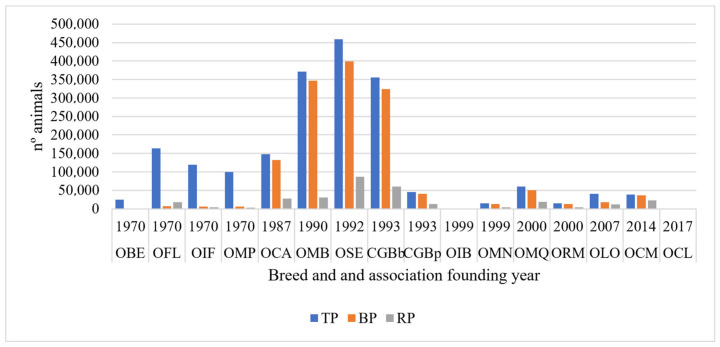
Total (TP), base (BP), and reference (RP) population sizes and foundation year of breeders’ association by breed.

The population sizes and foundation years of breeders’ associations for each breed are shown in Figure 2. Considerable variation can be observed among breeds, with the largest population corresponding to OSE (more than 450,000 animals in the TP), OMB, and CGBb (with TP sizes close to 350,000 individuals). Among the autochthonous breeds, these three have breeders’ associations established in the 1990s, as does OCA, although its census is considerably smaller (less than 150,000 animals in the TP).

Among the integrated breeds, whose association is the oldest (AECOP; Figure 2), the TP sizes are considerably smaller than those previously mentioned for OSE, OMB, and CGBb, with BP and RP each comprising fewer than 20,000 animals. OFL, OIF, and OMP display population sizes similar to OCA (TP ranging approximately from 100,000 to 170,000), while OBE shows the smallest population size within the integrated breeds’ group (fewer than 25,000 animals in the TP).

**Figure 3:**
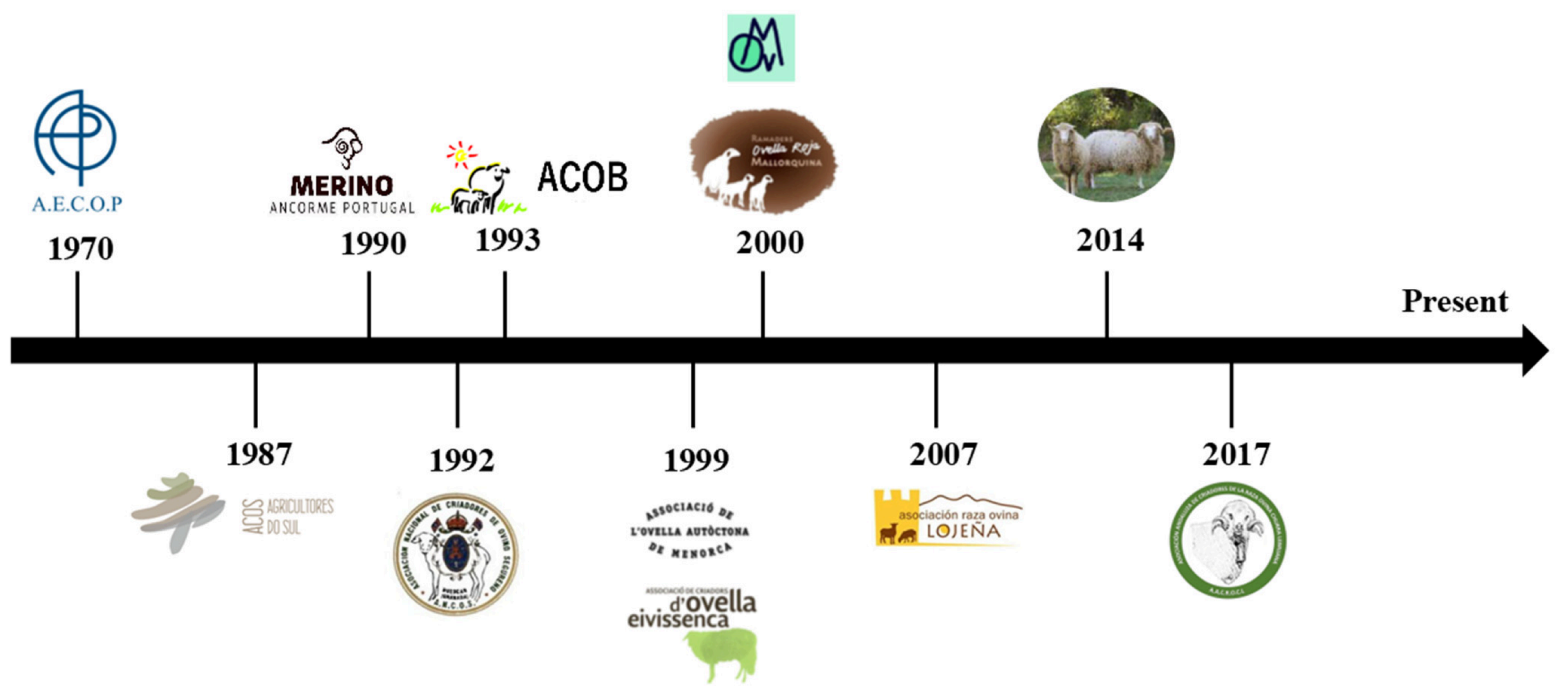
Year of foundation of breeders’ associations: Asociación Española de Criadores de Ovinos Precoces (AECOP), Associação de Agricultores do Sul (ACOS), Associação Nacional de Criadores de Ovinos da Raça Merina (ANCORME), Asociación Nacional de Criadores de Ovino Segureño (ANCOS), Associação Nacional dos Criadores de Ovinos da Raça Churra Galega Bragançana (ACOB), Associació De L’ovella Autóctona De Menorca, Associació De Criadors D’ovella Eivissenca, Associació Ovella Roja Mallorquina, Associació de Ramaders de l’Ovella de Raça Mallorquina, Asociación De Ganaderos Criadores De La Raza Ovina Lojeña Del Poniente Granadino (ACROL), Agrupamento de Produtores da Raça Ovina Churra Mondegueira (APROMEDA CRL), Asociación Andaluza de Criadores de la Raza Ovina Churra Lebrijana (AACROCL).

Larger population sizes than OBE were recorded for OMQ, CGBp, OLO, and OCM, with TP values ranging from 38,000 to 60,000 individuals, and showing very similar figures in their respective RP. The breeders’ associations of these breeds were established during the 2000s, except for CGBp, which belongs to the same association as CGBb. The smallest populations correspond to ORM (fewer than 15,000 animals), followed by OIB and OCL, whose TP barely exceeds 1.000 animals. The breeders’ associations of these three breeds were founded in 1999 (OIB) and in the 2000s (ORM and OCL), with that of OCL being the most recently created, in 2017 (AACROCL; Figure 3).

### 3.3. Extent of Farm Fragmentation Across Breeds

Table 1 shows the average farm sizes of all the breeds included in the present analysis. Breeds with smaller census sizes exhibited greater fragmentation of the RP. From highest to lowest fragmentation, these were: OIB, OMN, ORM, and OCL.

### 3.4. Number of Registered Sires

**Figure 4:**
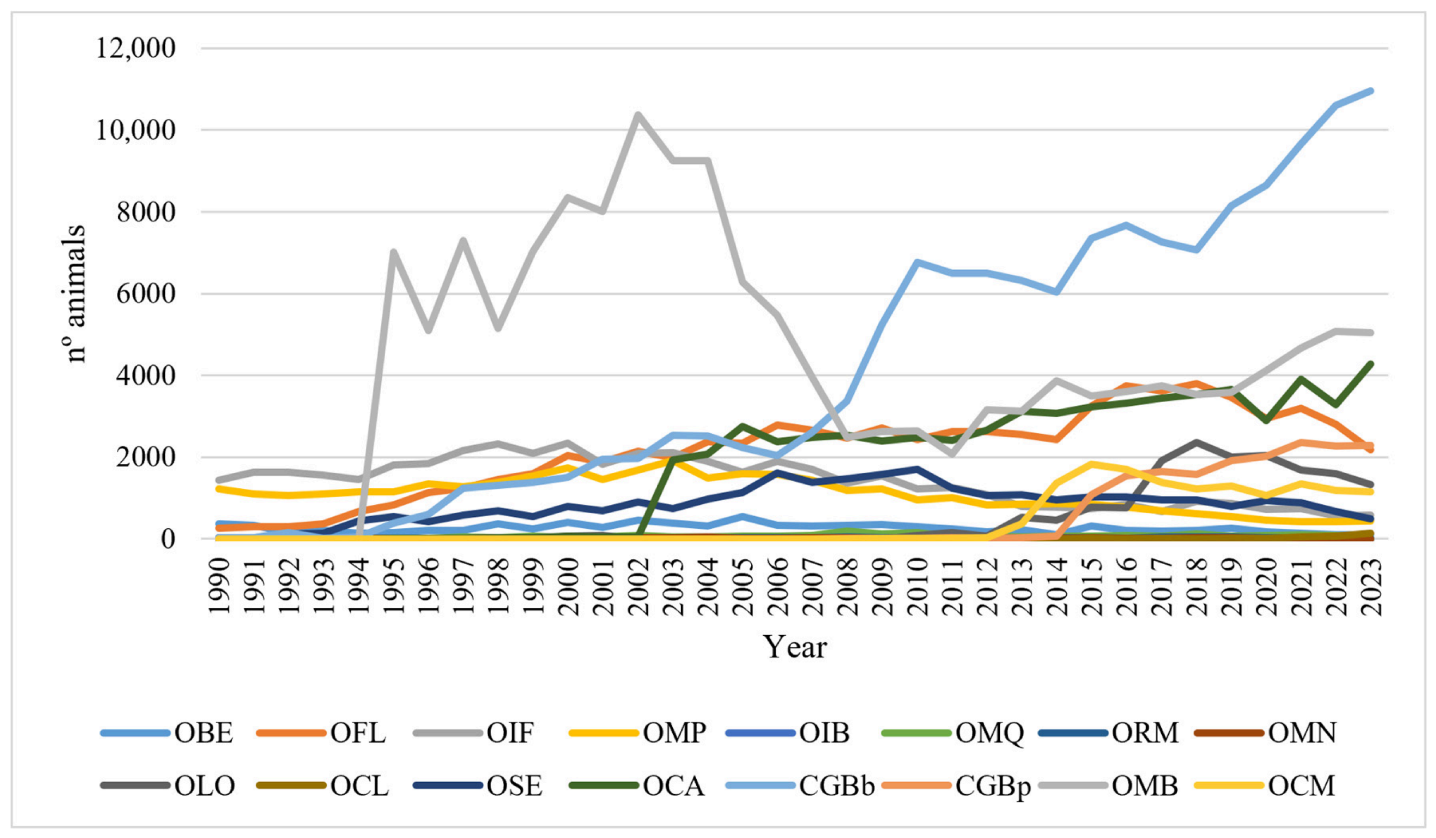
Annual trend in the number of born sires by breed.

Figure 4 presents the trends in sires born from 1990 onwards, highlighting two Portuguese local breeds, OMB and CGBb. OMB reached a peak of more than 10,000 registered sires in 2002, later stabilizing at lower levels (between 2000 and 5000 new registrations per year). CGBb has shown a gradual increase since the beginning of record keeping, exceeding 10,000 new registrations during the last two years. These are followed by OCA and OFL, which show comparable numbers of registrations (ranging from 2000 to 4000 sires over the past two decades). By contrast, while OFL records date back to before the 1990s, consistent record-keeping for OCA has only been observed since 2003.

Similarly, CGBp, OLO, and OCM have shown comparable growth trends since the beginning of their records (2013–2015), with CGBp exceeding 2000 new registrations in recent years, and OLO and OCM surpassing 1000.

Two other complementary figures (Supplementary Figures S1 and S2) highlight minor variations in breeds with smaller population sizes.

In the Spanish integrated breeds (Supplementary Figure S1), the high number of registrations observed for OFL has been previously noted. This breed is followed by OIF and OMP, which had higher registration numbers in 1990 but, together with OBE, have recorded fewer than 1000 sires per year over the last decade. Moreover, OBE has maintained low registration numbers throughout the study period.

The Spanish autochthonous breeds are presented in greater detail in Figure S2. Overall, the number of registrations is very low (below 2500). In contrast, OSE and OLO display more consistent recording patterns than the remaining breeds (OIB, OMQ, ORM, OMN, and OCL), all of which remain well below 500 new sire registrations per year.

### 3.5. Number of Registered Dams

**Figure 5:**
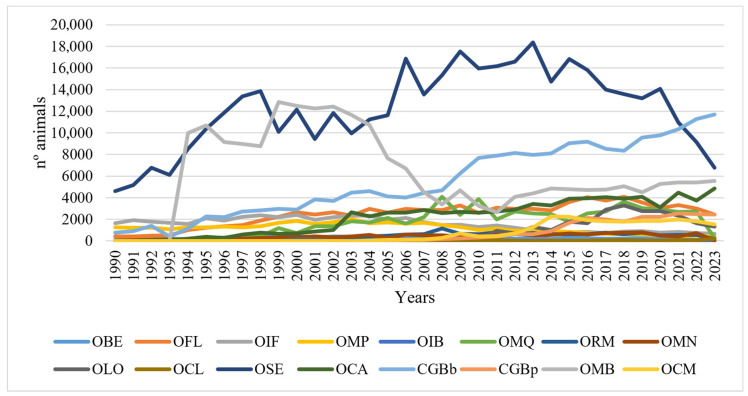
Annual trend in the number of born dams by breed.

For dams, OMB and CGBb once again stand out in comparison with the remaining breeds, except for OSE, which recorded the largest number of dams (over 10,000 new animals born annually from 1995 to 2022) until 2020, when numbers began to decline, falling below those of CGBb. CGBb exhibited a steady increase throughout the study period, while OMB peaked in births between 1994 and 2004 before decreasing and stabilizing at approximately 4000–6000 newborns per year. Likewise, OLO, OCM, and CGBp showed a marked rise between 2007 and 2010, followed by a decline and stabilization, although CGBp maintained a sustained upward trend (Figure 5).

Regarding the remaining breeds, a birth rate below 4000 dams per year is observed over time, except for OCA, which has shown a slight increase in 2023.

A closer view of Supplementary Figures S3 and S4 reveals the low number of animals born in OBE (fewer than 500 new dams per year), comparable to OCL and OIB. The remaining breeds range between 500 and 4000 births annually, with OFL showing the highest figures.

### 3.6. Parental Age at Reproduction

**Figure 6:**
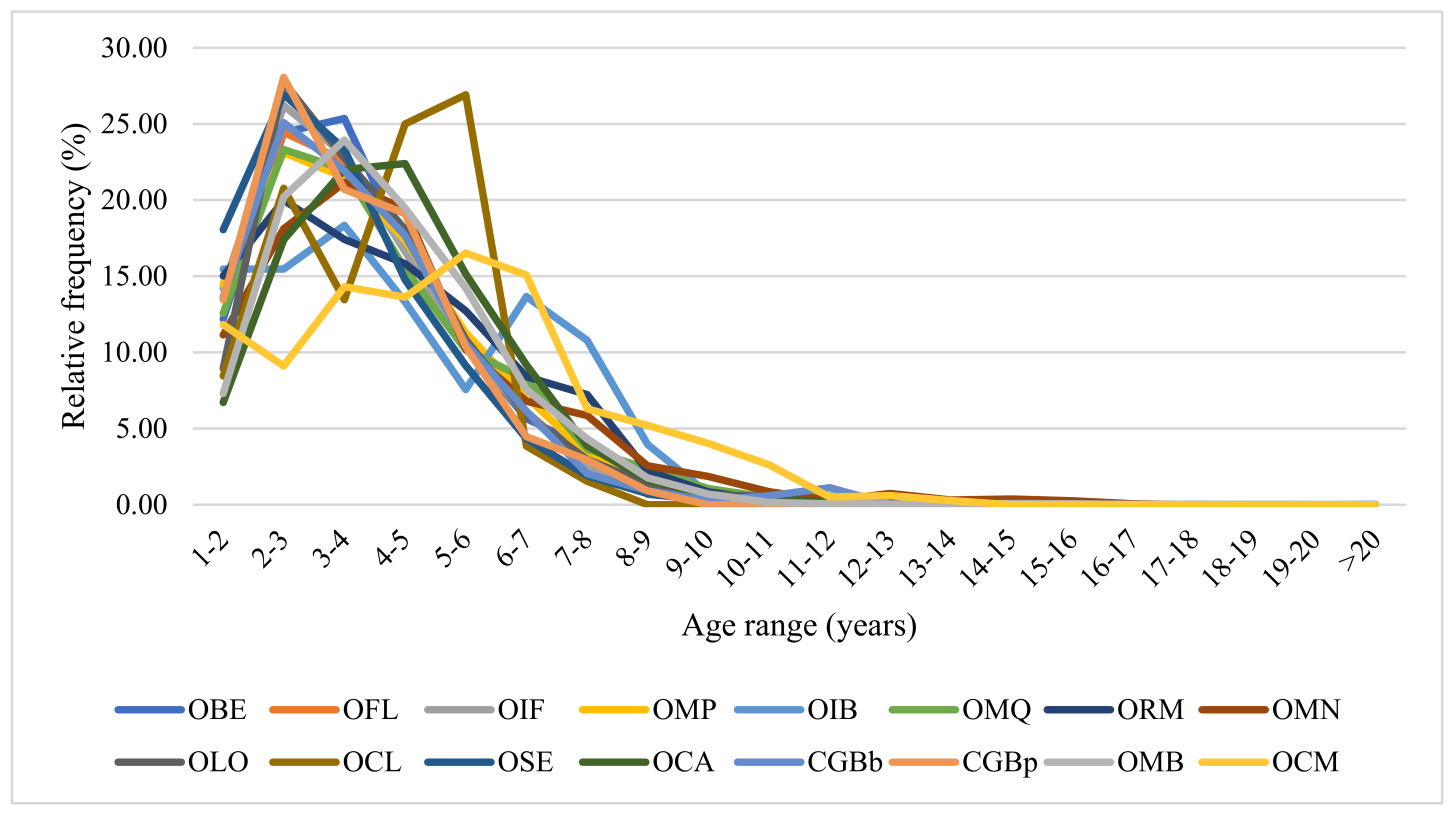
Age of sires in years when their registered offspring are born.

**Figure 7:**
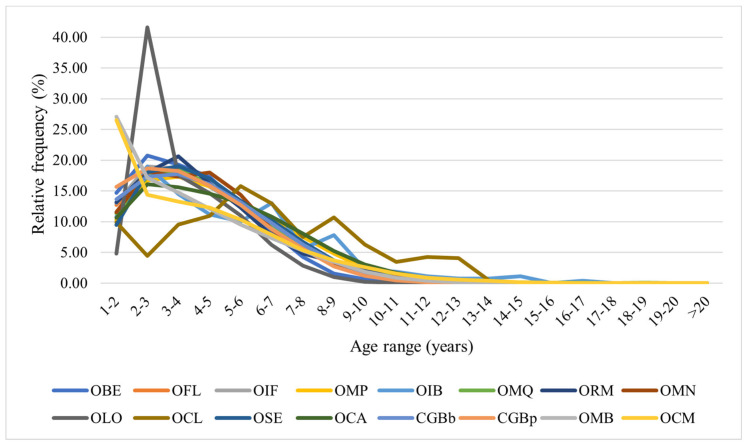
Age of dams in years when their registered offspring are born.

Sires and dams show similar age patterns at the birth of their offspring, with most individuals ranging from 1 to 7 years of age and a peak observed at 2–3 years (Figure 6 and Figure 7).

In general, all breeds exhibit similar age patterns, except for some autochthonous ones. Sires from most breeds reach their reproductive peak at 2–3 years of age (Figure 6). In contrast, in OCL and OCM this peak occurs later, at 5–6 years, and in OCA and OBE at 4–5 and 3–4 years, respectively. OIB sires display two distinct peaks, at 3–4 and 6–7 years, the latter being higher than the general trend and similar to that observed in OCM, whereas in most breeds the proportion of sires with offspring at this age falls below 10%. Remarkably, in OCM, instead of exhibiting a peak at 2–3 years (as seen in more than 20% of sires in other breeds), the frequency remains below 10%.

A comparable pattern is observed for the age of dams (Figure 7), in OCM, OMB and OCL. The latter lies well below the general trend, with only about 5% of dams producing offspring at 2–3 years of age, compared with more than 15% in the other breeds. Conversely, over 40% of dams in OLO have their offspring at 2–3 years of age, although this proportion declines sharply as dams age. Furthermore, OCL and OIB display secondary peaks associated with dams older than 5 years.

### 3.7. Offspring per Sire and Dam

In terms of reproductive performance, clear differences are observed between sires and dams, although some breeds deviate from the general trend.

**Figure 8:**
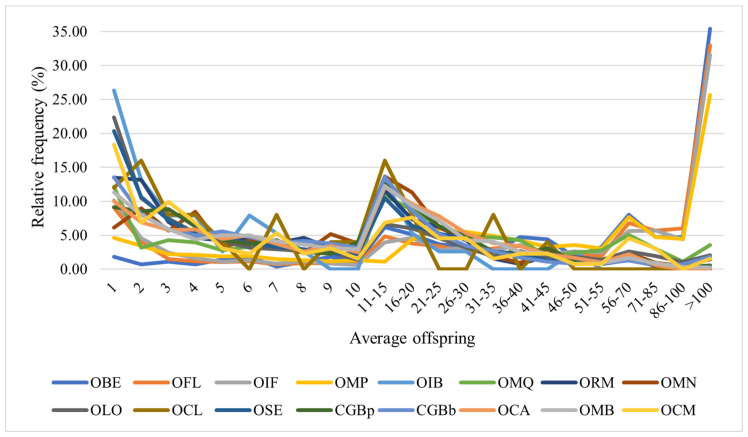
Relative frequency in the number of descendants per sire.

As shown for sires (Figure 8), the general pattern indicates a high proportion of sires with one offspring (5–25%) and with 10–25 offspring (5–15%). Integrated breeds differ from this pattern, showing lower proportions of sires in these categories (below 10%), while over 25% of sires have more than 100 offspring. Deviations from the general trend are mainly observed in OCL and OIB, which report proportions close to zero in several offspring ranges but still display a peak at 10–25 offspring per sire.

**Figure 9:**
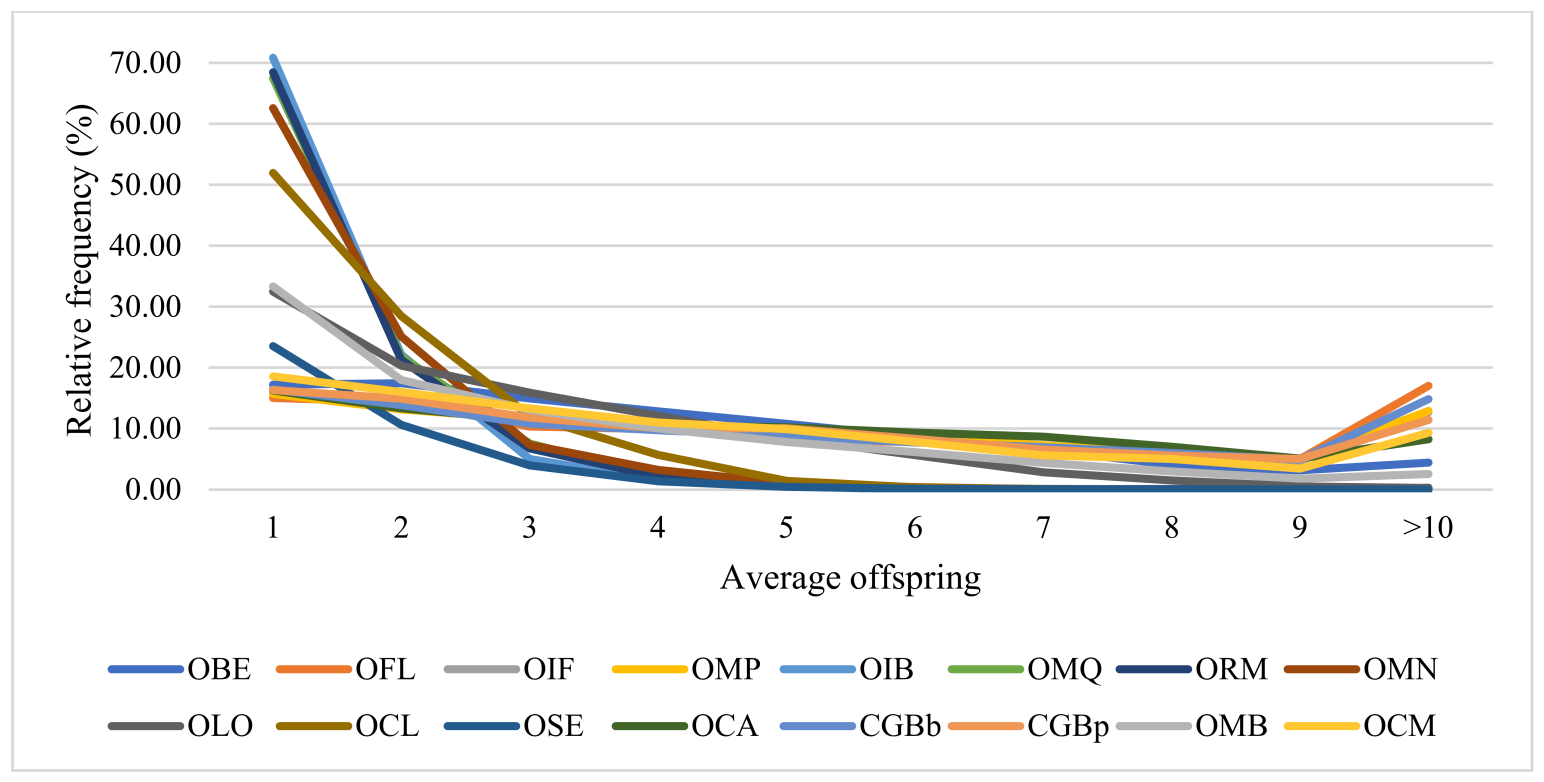
Relative frequency in the number of descendants per dam.

Two distinct trends can be identified in the reproductive performance of dams (Figure 9). The first trend is characterised by a high proportion of dams producing only one offspring, followed by a steep decline to 0% in those with five or more offspring. This trend is seen in the Spanish autochthonous breeds and OMB, with OIB, ORM, OMQ, OMN, and OCL showing over 50% of dams with a single offspring.

The second trend (Supplementary Figure S5) describes a gradual decline in the proportion of dams as the number of offspring increases, beginning with fewer than 20% of dams having a single offspring and showing a slight increase when the number of offspring reaches 10 or more. The highest proportions of dams within this trend are observed in OFL, CGBb, OMP, OIF, and CGBp, each with over 10% of dams producing 10 or more offspring. Additionally, not only do the aforementioned breeds follow this trend, but OMB and OLO also maintain a higher proportion of dams with five or more offspring.

### 3.8. Sex Ratio of Breeding Animals

**Figure 10:**
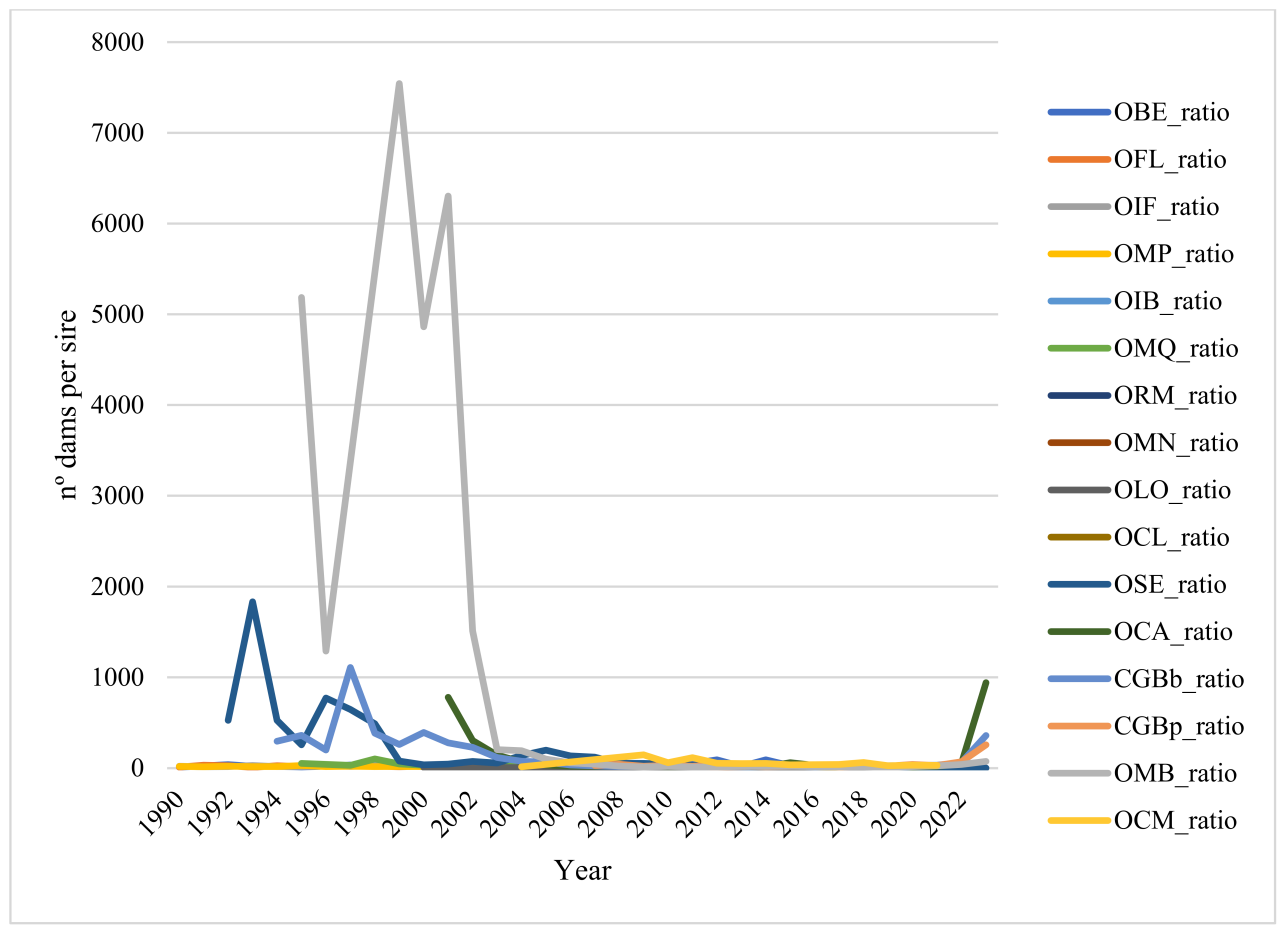
Annual sex ratio per year (number of females per male) among breeding animals of each breed.

Based on the annual number of breeding animals per breed (those with recorded offspring), the sex ratio was analysed from 1990 onwards (Figure 10), alongside the number of registered animals. In several breeds, notably OMB, OSE, CGBb, and OCA, exceptionally high sex ratio values were observed in some years, followed by an abrupt decline.

Supplementary Figure S6 illustrates the annual sex ratio in breeds with a lower proportion of females per male. Notably, CGBp shows an increase in its sex ratio during the final two years of the study period (2022–2023). The other breeds generally maintain a proportion below 50:1, although OMQ, OCM, and OBE exceed this threshold in isolated years within the study period.

### 3.9. Pedigree Completeness (PC)

**Figure 11:**
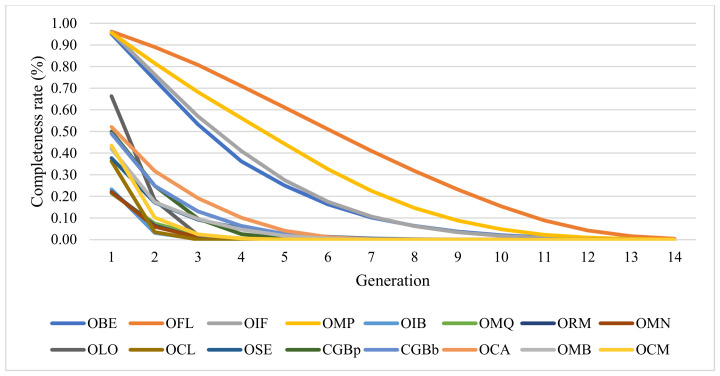
Average percentage of Pedigree Content (PC) per generation in Total Population (TP) of each breed.

Figure 11 shows the completeness rate of known ancestors across ascending generations for each breed. Integrated breeds exhibit a notably high pedigree completeness, reaching almost 100% in the first generation and experiencing only slight reductions in subsequent generations. This pattern is particularly evident in OFL, whose pedigree completeness remains above 20% through the tenth generation.

Among the autochthonous breeds, OLO exhibits the highest pedigree completeness in the first generation (66%), but also the steepest decline, with values dropping to nearly 0% by the third generation. A comparable reduction, reaching completeness rates below 10% in the third generation, is also evident in OSE and OCM, which display first-generation completeness rates of 38% and 44%, respectively.

For the remaining Portuguese breeds, pedigree completeness remains above 10% up to the fourth generation in OCA and the third generation in CGBp, CGBb, and OMB. In contrast, the Spanish autochthonous breeds (OIB, OMQ, ORM, OMN, and OCL) show completeness rates below 10% as early as the second generation.

**Figure 12:**
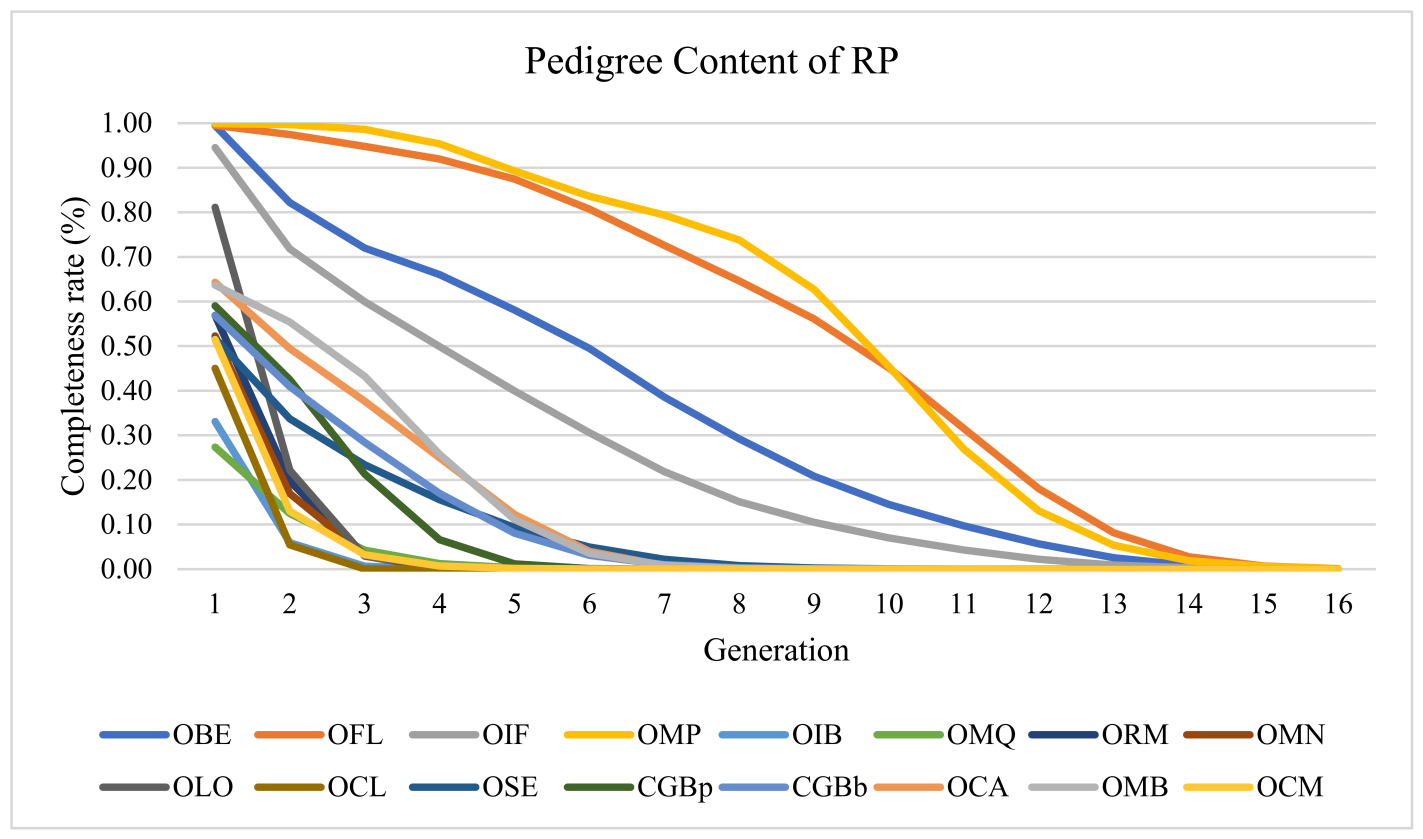
Average percentage of Pedigree Content (PC) per generation in Reference Population (RP) of each breed.

In general, pedigree completeness of the RP is higher (Figure 12). Integrated breeds once again display the highest values, particularly OFL and OMP, which maintain the most stable pedigree completeness through the twelfth generation, with rates of 18% and 13%, respectively.

As far as autochthonous breeds are concerned, a marked increase in OMB’s pedigree completeness is seen up to the fifth generation, followed by a gradual decline, similar to that observed in OCA and OSE, reaching values below 10% thereafter. The remaining breeds maintain completeness rates above 10% for one additional generation compared with the TP (OIB, OMQ, ORM, OMN, OCA, and CGBb), whereas OLO, OCL, OCM, and CGBp display comparable completeness levels.

### 3.10. Generation Interval (L)

Table 2 shows that most of the breeds studied present an L of approximately 4 years. Exceptions are OIB and OCL, whose L reaches 5 years, although in OIB this value is observed only for the RP. Slightly higher values than the trend are also recorded for OCA (4.58 and 4.46 for TP and RP, respectively), OCM (4.49 and 4.39), OMN (4.26 and 4.45), as well as in the RP of OMB (4.52).

## 4. Discussion

Demographic studies play a pivotal role in the management, conservation, and sustainable improvement of sheep breeds. These analyses provide essential information on population structure, temporal trends, genetic diversity, and reproductive dynamics, all of which are critical for ensuring the long-term viability of livestock populations [36]. In addition, this information reflects the ability to monitor different breeds and provides useful indicators for decision-making regarding population management. In the context of sheep breeding, demographic evaluations are particularly important because many breeds are characterised by relatively small effective population sizes, geographic isolation, or specialised production systems that increase their vulnerability to genetic erosion and population decline [37].

One of the primary objectives of demographic studies is to monitor changes in census size and breeding structure over time. Parameters such as the number of breeding males and females, generation intervals, replacement rates, and reproductive contributions allow researchers and breeding organisations to identify potential demographic bottlenecks [38]. A reduction in the number of active breeders or an excessive dependence on a limited number of sires causes bottlenecks and, consequently, is likely to accelerate the loss of genetic variability and increase inbreeding levels. Such processes can compromise fitness, reproductive performance, adaptability, often associated with inbreeding depression, and disease resistance, ultimately threatening breed sustainability [39].

Demographic analyses are also essential for evaluating the effectiveness of breeding and conservation programs. Many local and autochthonous sheep breeds are maintained under conservation schemes due to their cultural, ecological, or productive value. These breeds often possess unique genetic adaptations to harsh environments, low-input production systems, or endemic diseases. Nevertheless, without continuous demographic monitoring, conservation efforts might fail to prevent gradual population fragmentation or hidden declines in effective population size. Demographic indicators, therefore, provide an evidence-based framework for designing mating strategies, optimising sire usage, and maintaining balanced genetic contributions among flocks [40].

Furthermore, demographic studies contribute to understanding the historical development and management practices of breeds. Temporal analyses of pedigree depth and registration patterns can reveal periods of expansion, selection intensification, or changes in breeding policies. This information is valuable for interpreting current genetic diversity levels and predicting future trends. In commercial breeds, demographic assessments support genetic improvement programs by helping maintain sufficient diversity while maximizing selection response [41]. In endangered populations, they guide prioritization decisions and facilitate the implementation of cryoconservation or assisted reproduction strategies when necessary [42].

### 4.1. Trends in Integrated Breeds

As a whole, there is considerable variability among breeds for each parameter; however, certain differences in their trends should be highlighted.

Despite their moderate census size, integrated breeds exhibited the most complete pedigree records, suggesting a more consolidated breeding management and systematic pedigree recording than that observed in most autochthonous breeds. Moreover, the observed pattern characterized by a high number of offspring per sire and dam (second-trend breeds), alongside low annual registrations (fewer than 4000 sires and dams per year). could be interpreted, on the one hand, as an intensive use of a limited number of males through Artificial Insemination (AI) within the breed, potentially suggesting a demographic risk [43]. Conversely, it may result from the extensive use of sires producing large numbers of semen doses primarily intended for crossbreeding and improvement programs for native breeds, meaning that much of their progeny is not recorded in their own herdbook. In this study, such progeny is not included, as the database corresponds solely to the breed’s herdbook. Therefore, additional genetic diversity parameters, such as inbreeding coefficients or the effective number of founders and ancestors, would be required to determine whether a genuine loss of genetic diversity exists.

Some of these breeds have been included in genetic diversity studies involving breeds from different countries, or conducted in their countries of origin, such as OBE [22], in which a mean L of 4.10 was reported (slightly higher than the value obtained in our study, 3.75). This study also described a good pedigree depth, assessed through the cumulative percentage of dams born during the study period (1996–2000) with both parents known.

### 4.2. Pedigree Completeness and Data Reliability in Autochthonous Breeds

In this context, the low PC observed in autochthonous breeds is particularly noteworthy, revealing deficiencies in pedigree recording as a result of the management practices used, especially regarding paternal information. This leads to an underestimation of genetic diversity parameters based on multiple ancestors, such as inbreeding (F), the genetic conservation index (GCI), and effective population size (Ne). Improving pedigree completeness in these breeds is essential to ensure reliable analyses and robust genetic evaluations. The higher pedigree completeness observed in most Portuguese breeds likely reflects more consistent pedigree recording and long-term herdbook management, except for OCM, whose breeders’ association was only recently established (2014) and has been making an effort to verify parentage through DNA testing.

Differences in pedigree completeness among breeds should also be considered when comparing demographic parameters, as estimates derived from populations with less complete genealogies could be more affected by incomplete ancestral information. Nevertheless, the main demographic indicators analysed in this study (e.g., annual registrations, parental age, sex ratio and generation interval) are based primarily on recorded reproductive events and are, as a result, less sensitive to pedigree depth than pedigree-derived genetic parameters such as inbreeding or effective population size.

Regarding pedigree records, breeds with larger census sizes generally show higher numbers of registered sires and dams, except for OSE sires, whose figures do not exceed 1700 sires per year. OSE is a highly productive breed, classified as a ‘promotional breed’, partly due to the widespread use of reproductive technologies such as AI, which are not commonly applied in other local breeds reared under more traditional production systems [44,45,46]. For this reason, OSE is expected to be more efficient in reproductive terms, reducing the need for a large number of breeding sires while prioritizing the production of lambs for commercial purposes. This is also reflected in the OSE sex ratio, which exceeds 50 dams per sire between 2002 and 2009 (values before this period are higher, probably due to incomplete registrations in the early years of the breeders’ association). From 2010 onwards, even so, a decline in the sex ratio can be observed. During the aforementioned period, OSE exhibits the highest sex ratio among the Spanish breeds (including the integrated ones) while the Portuguese local breeds also present high sire efficiency (values exceeding ten dams per sire).

In addition, OSE and the Portuguese breeds show similarly low PC values (higher than those of the Spanish endangered breeds but still insufficient for estimating deep pedigree-based parameters with confidence). This low PC observed in OSE has also been reported in previous research [20], in which an underestimation of genetic diversity parameters, such as inbreeding and average relatedness, was noted. The mean L reported in that study for OSE (3.79) is lower than the value obtained in the present work (4.20).

### 4.3. Annual Registrations and Sex Ratio Dynamics

The high female-to-male ratios observed in OMQ, ORM and OMN cannot be interpreted in the same way as in commercial breeds, because several demographic and recording-related factors may contribute to this pattern. First, although AI could partially account for this pattern, its use is not widespread in these breeds. Second, the results could be interpreted as an excessive use of sires driven by commercial interests, or in other words, an imbalance between male lambs kept for breeding and those destined for sale. Nevertheless, the most plausible explanation is the low pedigree completeness, which is below 30% in the TP (approximately 21–22% and 6–7% in the first and second generations, respectively). This is presumably due to a high number of dams with unknown sires. Such under-registration of males is consistent with the low PC observed. Consequently, when the number of registered females increases while the number of registered males does not, the sex ratio becomes artificially inflated. This premise is particularly evident in the case of the OMB, in the years from 1995 to 2001. During the early years following the association’s foundation, there was an extremely high rate of animal registration and enrolment, which caused the sex ratio to become unbalanced. In any case, it would be valuable for future demographic analyses to also consider the proportion of progeny selected for breeding, as proposed by Casanovas Arias et al. [3].

### 4.4. Productivity and Longevity Patterns

A similar trend, characterised by generally long productive lifespans, was observed in the breeds studied. However, the exceptions are OBE and OLO, with values of 2.40 and 1.29% for the aforementioned variable, respectively.

In the case of OCL and OIB breeds, their critical risk status means that the few existing breeding animals are primarily used to preserve the breed itself. To support this objective, reproductive techniques such as AI are used in OIB to increase the number of descendants [47], and in OCL as part of a broader effort to reconstruct the breed [48]. This situation is also reflected in their L values, which are higher in these breeds (4.55 in OIB and 4.95 in OCL). The relatively large standard deviations observed for the generation interval indicate considerable variability in the age at which breeding animals are replaced within populations. This variability probably reflects differences in replacement strategies among flocks and the coexistence of young and older breeding animals. In some endangered breeds, such as OIB and OCM, the highest standard deviations can also be associated with the prolonged reproductive use of selected individuals due to the limited availability of breeding animals.

Despite being classified as endangered, OLO has a larger population than that of other local Spanish breeds (11,606 animals in the RP), comparable only to OMQ (18,827 animals in the RP). Although this breed does not appear to be particularly long-lived (90.92% of dams produce offspring between 2 and 7 years of age), it is the most productive among the local Spanish breeds, with 18.43% of dams having between five and eight descendants. Nonetheless, this breed is managed under extensive and ecological production systems, without any specific reproductive management practices, and its prolificacy (1.09) is lower than that of the other breeds studied [49]. This indicates that its productivity does not depend on longevity or prolificacy, but rather on a very consistent reproductive performance during a relatively short productive lifespan [50,51]. The relatively short productive life of breeding animals might be explained by the hypothesis, based on breeders’ knowledge, that rapid tooth wear occurs due to environmental characteristics in which forage height is very low [52]. Under this hypothesis, the lower L observed in OLO (3.82) compared with other local breeds is consistent, although this remains unproven.

Considering the average offspring per dam, the autochthonous Portuguese breeds follow the same trend as the integrated breeds, exhibiting higher productivity than the other local breeds. Portuguese breeds are managed under extensive production systems with one lambing per year, as reported in the demographic analyses of Carolino et al. [26,27,28,29,53]. This pattern seems to indicate either a long productive lifespan or a moderate-to-high prolificacy. AI could also contribute to this pattern, as its use has been documented in OCA and OMB [54]. Alternatively, this parameter likely reflects differences in the efficiency and completeness of pedigree recording systems among breeds. Such systems involve multiple stages, including on-farm identification and reporting of births by breeders, field verification and data collection conducted by breed associations, and the subsequent integration and digitization of herdbook records. Breeds supported by more robust recording infrastructures and data management procedures are expected to exhibit higher pedigree completeness and data quality, thereby improving the reliability of genealogical information and related genetic analyses [40]. Moreover, considering their L values, their replacement pattern appears to be approximately four years, similar to the other breeds. Despite this, sires do not display the same high number of descendants. This asymmetry is expected in populations with low PC, in which dams tend to be systematically registered while many sires remain unrecorded.

Productive longevity has become an increasingly important objective in livestock breeding programs due to its close relationship with resilience, animal welfare, and environmental sustainability. In sheep populations, extending the productive life of breeding animals contributes not only to improving farm profitability but also to enhancing the adaptive capacity and long-term viability of breeds [55,56]. Animals capable of maintaining reproductive and productive performance over several years are generally better adapted to their production environments and more resistant to health, nutritional, and climatic challenges. Consequently, longevity can be considered an indirect indicator of functional robustness and resilience [57].

From an economic perspective, extending productive lifespan reduces replacement costs and improves resource-use efficiency, as fewer young animals are needed to maintain flock size. In addition, animals with longer productive careers distribute the environmental burden associated with rearing replacement stock over a greater amount of output, thereby reducing greenhouse gas emissions per unit of product [58]. This is particularly relevant in the context of sustainable livestock production, since improving environmental performance has become a key objective in response to increasing societal and policy demands.

### 4.5. Demographic Risks and Genetic Consequences in Autochthonous Breeds

Low-population Spanish autochthonous breeds (OIB, OCL, OMQ, ORM, and OMN) exhibit higher demographic risks, characterized by low PC, low annual numbers of registered breeding animals, and an intensified use of sires. This overuse of sires is reflected both in the age at which their offspring are born, particularly evident in OIB, in which more than 10% of sires are at least seven years old, and in the distribution of descendants, with OMQ showing 15.49% of sires having more than 50 offspring. Additionally, OIB and OCL show higher L values (approximately 5 years, one more than the overall average), which can be attributed to the extended use of breeding animals. From a breeding program perspective, reduced generation intervals (L) are generally desirable to increase annual genetic gain and economic efficiency [20,59]. OCL, in particular, exhibits a marked overuse of dams, with 20.4% producing offspring at 8–11 years of age.

These factors could lead to genetic drift due to the disproportionate use of a limited number of breeding animals and, consequently, to a loss of genetic diversity. It is also important to consider the possible formation of subpopulations in breeds in which intensive use of a few individuals coincides with limited gene flow among herds [60]. Thus, by incorporating pedigree-based studies to control for demographic and diversity effects in small ovine populations, breeders’ associations, together with their geneticists, can develop functional tools that ensure the future viability of these genotypes. From a genetic perspective, demographic bottlenecks occur when only a limited number of individuals contribute offspring to subsequent generations. This reduction in effective population size increases the effects of genetic drift, reduces genetic diversity, and may lead to higher levels of inbreeding [61].

In fact, genetic signatures of a demographic bottleneck in OCL have been reported in another study, in which Calvo et al. [20] evaluated the intra-breed genetic variability of this breed. Moreover, the intensive use of a limited number of breeding sires observed in OMQ is also documented in the study by Goyache et al. [62], which reported evidence of genetic bottlenecks (based on the same herdbook database available in 2006) resulting in the appearance of inbred animals but with no apparent impact on overall genetic diversity.

### 4.6. Role of Breeders’ Associations and Population Fragmentation

Finally, the role of breeders’ associations is essential for managing the evolution of all breeds and indispensable for the development of breeding programs (whether aimed at improvement, conservation, or reconstruction). The coordination among the different affiliated farms makes these efforts feasible. For this reason, population fragmentation for each breed was also considered in our results (Table 1). Based on census data (Figure 1), one might expect OSE or even CGBb to be the breeds with the largest number of active breeders. However, several breeds with very small census sizes also show a high number of breeders, such as OIB, ORM, and OMN, resulting in elevated fragmentation ratios (7.88, 63.95, and 45.46 animals per farm, respectively). The RP of OIB is notably fragmented, likely due to its traditional use as family livestock, in which each farm maintained a very small flock [21].

The geographical dispersion and fragmentation of breed populations can hinder the effective implementation of breeding and conservation programmes, as breeders’ associations face greater challenges in monitoring population structure, maintaining accurate records, and coordinating management actions across all herds [63,64].

Parentage verification remains a major challenge for extensively managed sheep farms. While the social and environmental advantages of extensive systems are evident, current labour shortages and the high costs associated with molecular or genomic analyses do not offer a practical solution for parental control, which is essential for the efficient general management of the Iberian sheep population.

The importance of breeders’ associations has also been highlighted in previous studies [20,63], together with the value of cryopreservation programs for rare breeds [19] and the need to maintain long-term pedigree information to monitor changes in genetic diversity [38].

Several studies on genetic diversity in France have examined different rare breeds within the same geographical context [19,35]. Danchin-Burge et al. [19] in particular, evaluated the evolution of the number of breeding animals by comparing different birth-year cohorts, an approach that would be valuable to incorporate in future demographic studies. Other research has also demonstrated the rich reservoirs of genetic diversity present in some of the breeds included in the current study, such as OCL [21].

## 5. Conclusions

This demographic analysis of sixteen ovine breeds from the Iberian Peninsula provides a comprehensive overview of their population structure, reproductive patterns, and pedigree quality, revealing clear variations between breeds. The results highlight the need to reinforce systematic pedigree recording, particularly in endangered populations in which parental information is frequently lacking. The distribution of offspring per dam in Portuguese local breeds suggests longer productive lifespans, moderate-to-high prolificacy or highly consistent reproductive performance. Spanish endangered breeds exhibited the most fragile demographic structures, indicating increased risks of genetic drift, bottlenecks and the formation of subpopulations, while OLO stood out for its consistent reproductive output concentrated within a relatively short reproductive period. Together, these findings provide a solid basis for future genetic diversity assessments and support the development of conservation and breeding programmes for Iberian ovine breeds. Taken together, our results underscore the importance of coordinated breed management to safeguard the genetic heritage of Iberian ovine breeds in the face of evolving production and environmental contexts.

## Figures and Tables

**Table 1:** Average farm size of each studied breed.

Breed	Nº Active Breeders	RP	RP/nº Farms
OBE	7	897	128.14
OFL	32	17.833	557.28
OIF	15	4087	272.47
OMP	15	2994	199.60
OIB	24	189	7.88
OMQ	103	18.827	182.79
ORM	58	3709	63.95
OMN	85	3864	45.46
OLO	36	11.606	322.39
OCL	7	611	87.29
OSE	218	86,563	397.08
OCA	64	27.680	432.50
CGBb	155	59.955	386.81
CGBp	56	12.597	224.95
OMB	55	30.281	550.56
OCM	30	22.859	761.97

Abbreviations: RP = reference population (animals alive in 2023); RP/nº Farms = reference population divided by the number of active farms in 2023.

**Table 2:** Mean Generation Intervals (L) and Standard Deviation (SD) of Total (TP) and Reference Population (RP) for each breed.

Breed	TP	RP
OBE	3.75 ± 1.82	4.13 ± 2.08
OFL	3.92 ± 2.03	3.94 ± 1.96
OIF	3.88 ± 1.92	3.99 ± 1.93
OMP	4.18 ± 2	4.23 ± 2.11
OIB	4.55 ± 2.84	5.52 ± 3.16
OMQ	3.86 ± 2.09	3.91 ± 1.98
ORM	4.1 ± 2.13	4.09 ± 2.18
OMN	4.26 ± 2.27	4.45 ± 2.35
OLO	3.82 ± 2.39	4.09 ± 2.47
OCL	4.95 ± 2.07	5.08 ± 2.13
OSE	4.2 ± 2.01	4.24 ± 1.99
CGBp	3.78 ± 1.77	3.82 ± 1.75
CGBb	3.94 ± 1.92	3.88 ± 1.85
OCA	4.58 ± 2.15	4.46 ± 2.10
OMB	4.08 ± 2.20	4.52 ± 1.58
OCM	4.49 ± 2.77	4.39 ± 2.36

## Data Availability

The pedigree datasets analysed in this study were provided by the respective breeders’ associations responsible for the official herdbook of each breed. These data are not publicly available but may be available from the corresponding author upon reasonable request and with permission of the breeders’ associations.

## Supplementary Materials

The following supporting information can be downloaded at: https://www.mdpi.com/article/10.3390/biology15151233/s1, Figure S1: Annual trend in the number of registered sires in integrated breeds; Figure S2: Annual trend in the number of registered sires in Spanish autochthonous breeds; Figure S3: Annual trend in the number of registered dams in integrated breeds; Figure S4: Annual trend in the number of registered dams in low-population autochthonous breeds; Figure S5: Relative frequency distribution of offspring number per dam in breeds showing a high proportion of dams with large progeny size; Figure S6: Annual sex ratio (number of females per male) among breeding animals in breeds with sex ratio values below 260:1.

## Abbreviations

The following abbreviations are used in this manuscript:

AI	Artificial Insemination
BP	Base Population
CGBb	Churra Galega Bragançana Branca
CGBp	Churra Galega Bragançana Preta
F	Inbreeding Coefficient
GCI	Genetic Conservation Index
L	Generation Interval
MAPA	Ministerio de Agricultura, Pesca y Alimentación (Spain)
Ne	Effective Population Size
OBE	Berrichon du Cher
OCA	Campaniça
OCL	Churra Lebrijana
OCM	Churra Mondegueira
OFL	Fleishcschaf
OIB	Ibicenca
OIF	Île de France
OLO	Lojeña
OMB	Merino Branco
OMN	Menorquina
OMP	Merino Precoz
OMQ	Mallorquina
OSE	Segureña
PC	Pedigree Completeness
RP	Reference Population
TP	Total Population
