# Supplementary material for: Demographic Analysis of Sixteen Iberian Sheep Breeds Based on Pedigree Data
Source: Biology (Basel). 2026 Jul 24;15(15):1233. doi: 10.3390/biology15151233 (PMC13464712; doi:10.3390/biology15151233)
Supplement: Supplementary file 1 [file biology-15-01233-s001.zip › biology-4444725-supplementary.pdf]

Supplementary Material. ***Demographic analysis of sixteen Iberian sheep breeds based on pedigree data***

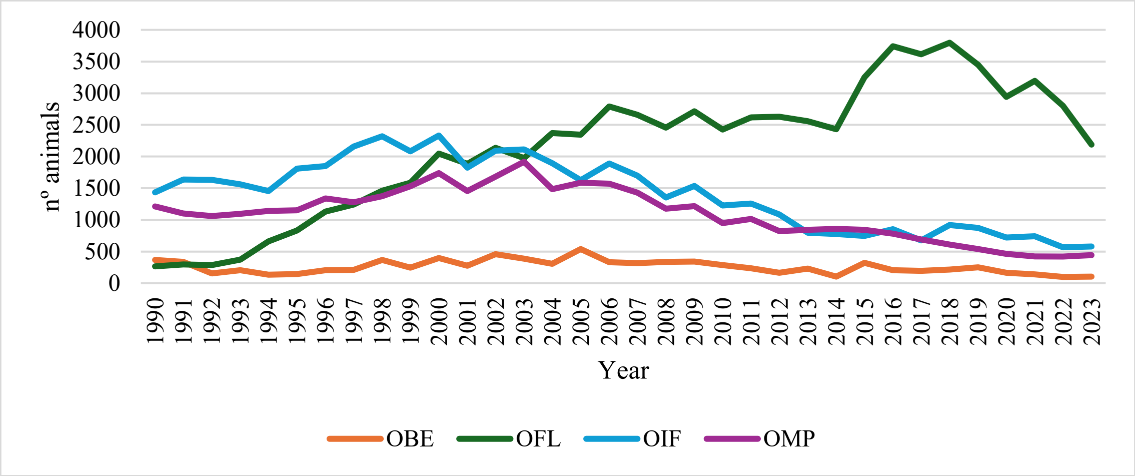

Figure S1. Annual trend in the number of registered sires of integrated breeds

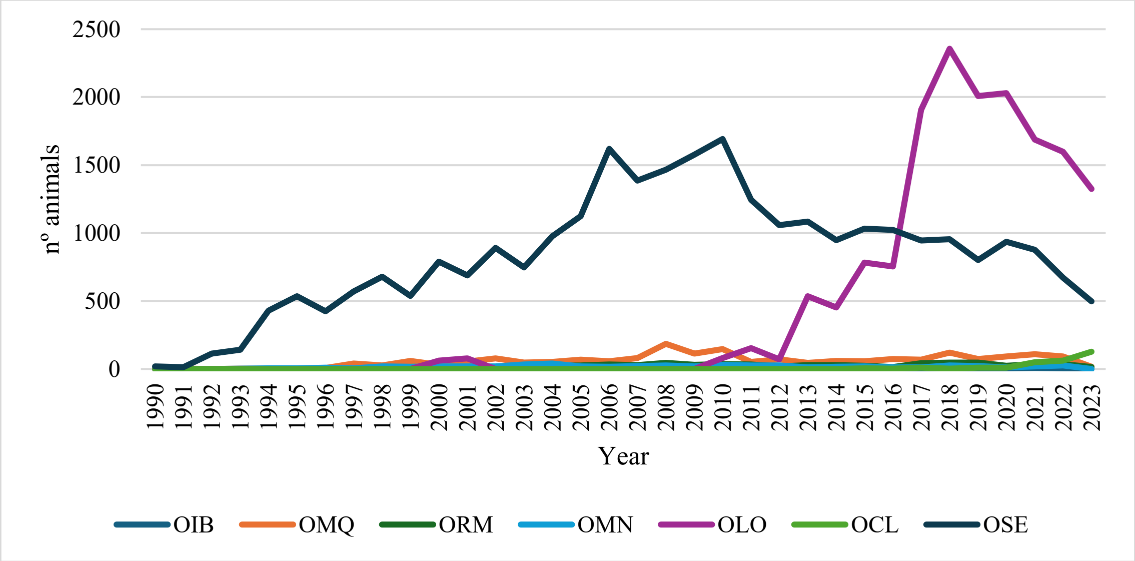

Figure S2. Annual trend in the number of registered sires of autochthonous Spanish breeds

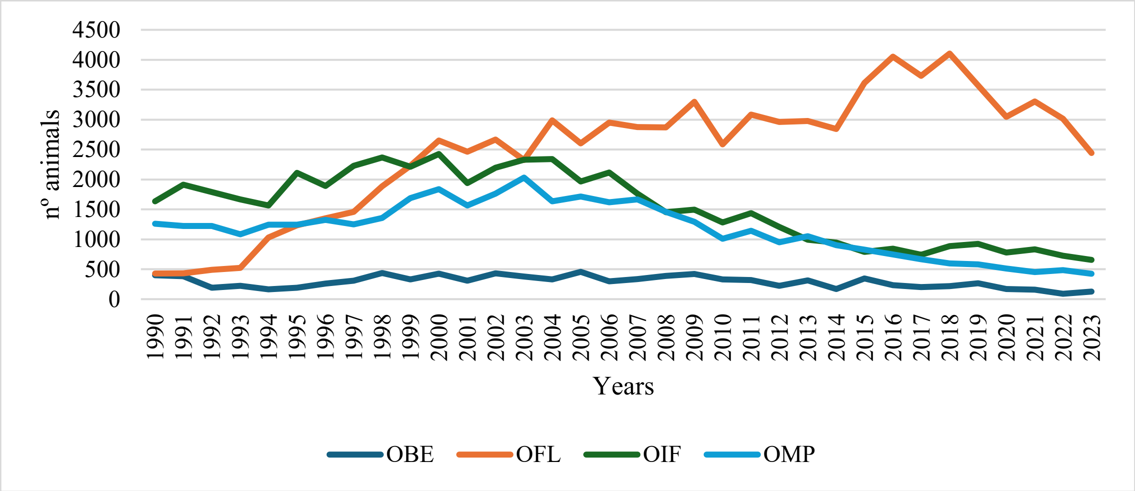

Figure S3. Annual trend in the number of registered dams in integrated breeds

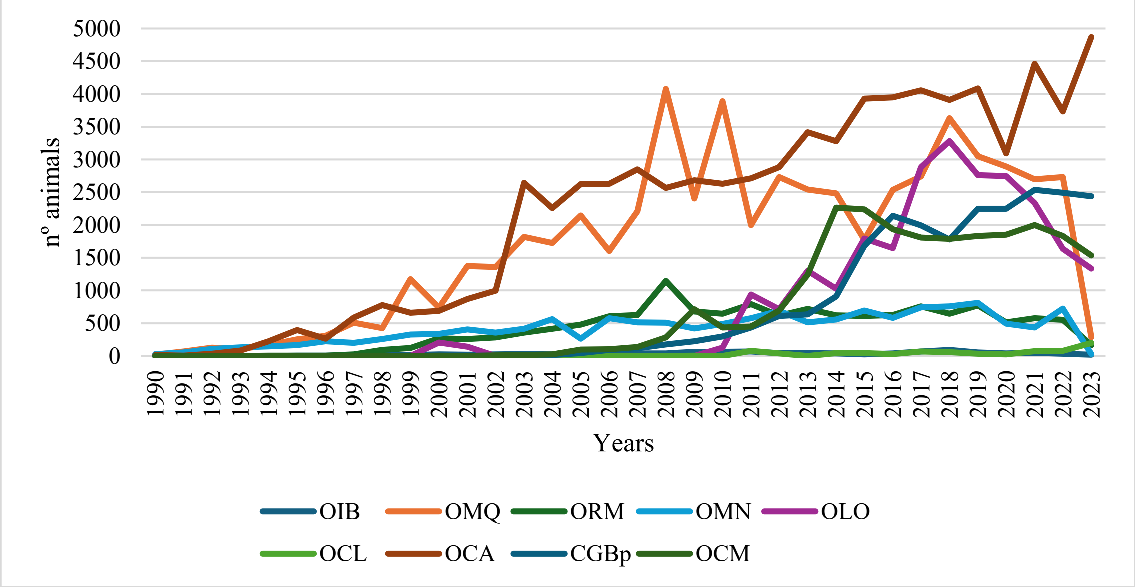

Figure S4. Annual trend in the number of registered dams in low-population autochthonous breeds

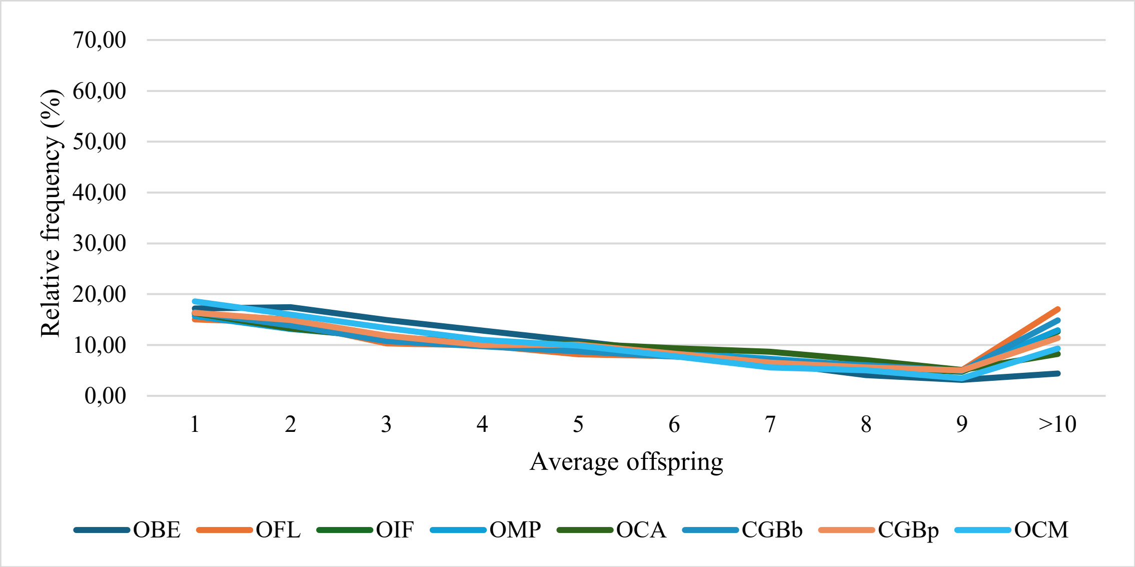

Figure S5. Relative frequency in the number of descendants per dam in breeds showing a high proportion of dams with a larger number of descendants

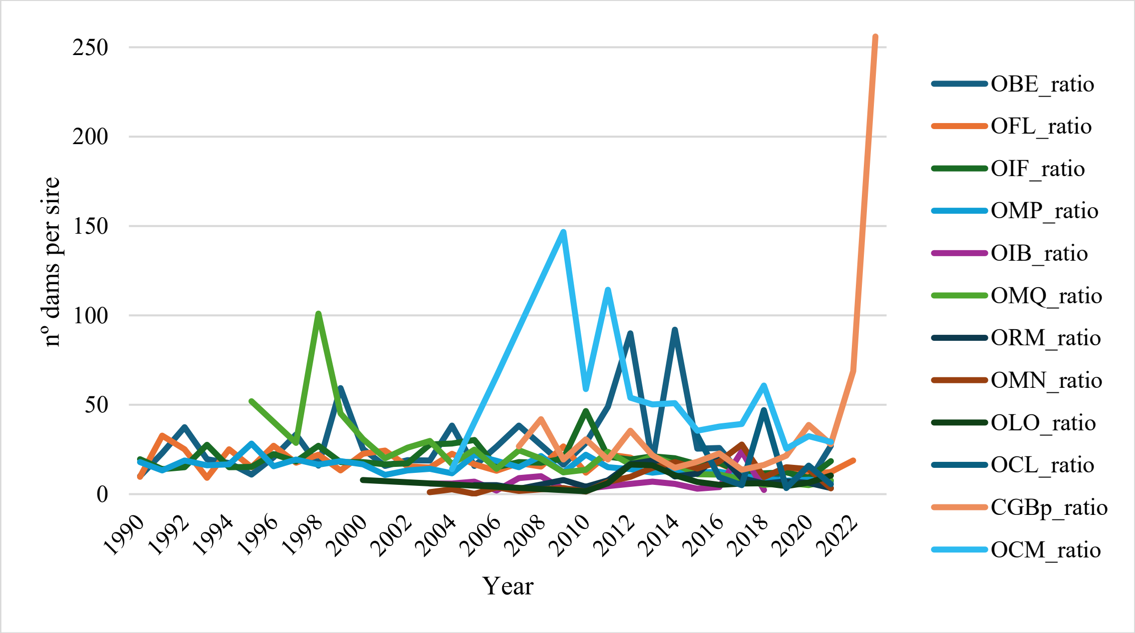

Figure S6. Annual sex ratio (number of females per male) among breeding animals of breeds with values below 260:1
